# Results of Kidney Transplantation from Donors Following Euthanasia in the Netherlands: Benchmarking Science and Ethical Challenge

**DOI:** 10.3389/ti.2024.13806

**Published:** 2024-10-18

**Authors:** Nichon E. Jansen, Dale Gardiner

**Affiliations:** ^1^ Department of Donation Policy, Dutch Transplant Foundation, Leiden, Netherlands; ^2^ Deceased Organ Donation, NHS Blood and Transplant, Bristol, United Kingdom

**Keywords:** transplantation, organ donation, euthanasia, ethical challenges, DCD-V

## Introduction

Susanna et al. report the 10 years follow-up results of kidney transplantation in the Netherlands from donors following euthanasia [1]. These are patients who following the legal administration of euthanasia under Dutch law, proceed to donation after circulatory death (DCD-V). They compared this group to the outcomes over the same period in controlled DCD (DCD-III) and donation after brain death (DBD). Uncontrolled DCD (DCD-II), donation after cardiac arrest in a brain dead patient (DCD-IV) and living kidney donation were excluded. The authors found from their study that the graft results of these kidneys have less delayed graft function than DCD-III donors and at least comparable longitudinal eGFR and graft function over 10 years as compared to kidneys from DCD-III and DBD. Their conclusion was that “…these results support the concept that ODE kidneys are a promising contribution to the donor pool, *and organ donation after euthanasia (ODE) should be continued*”.

Their paper raises two aspects of particular interest to the international donation and transplantation community – benchmarking science and ethical challenge.

## Benchmarking Science

The publication of outcome data and process metrics by countries is helpful because it allows other countries who do similar, or wish to do, to have a benchmark to compare against. The finding by Susanna et al. that delayed graft function was less in their DCD-V cohort than their DCD-III seems at face value, physiologically self-evident. DCD-III patients typically have had a devastating brain injury and a prolonged ICU stay before donation occurs. While the reasons for euthanasia in the DCD-V cohort were not provided in the paper, they are typically related to either neurodegenerative or psychiatric disorder in the Netherlands. Another expectation might be that warm ischemia time would be longer in DCD-III. This however was not the case and no difference was seen.

The comparable longitudinal eGFR and graft function over 10 years compared to both DCD-III and DBD is very reassuring and certainly gives credence to the authors’ claim that – medically at least – DCD-V kidneys are a safe extension of the donor pool.

## Ethical Challenge

Just because you can does not mean you should. For many countries in the world, even those with long established deceased donation programs, DCD-V is a step too far. And yet, there is a growing number of advanced economy countries who have, in the last decade, legalized euthanasia. Once they do, whether intended or not, DCD-V follows soon after. Belgium introduced a euthanasia law in 2002 and performed the world’s first DCD-V in 2005. The Netherlands legalized euthanasia in 2001, followed by the first DCD-V in 2012. In Spain medical assistance in dying became legal in June 2021, and by 2022, 4.6% of their DCD cases were DCD-V; which exceeds their long established DCD-II program (2.6%) [2]. The State of Victoria, Australia, introduced voluntary assistance in dying in 2019, they had their first DCD-V case in 2023 [3]. Other States in Australia have followed. In Canada, medical assistance in dying became legal in 2015, and Quebec carried out its first DCD-V in 2017. By 2022 DCD-V represented 14% of Quebec’s total deceased donation activity [4].

In the Netherlands, 15% of all DCD donors were DCD-V donors in 2023, which, like Quebec, is a substantial contribution to the donor pool. However, that does not mean that in all hospitals in the Netherlands DCD-V is facilitated. A small number of hospitals remain against euthanasia, for example, due to religious beliefs. Also, not all general practitioners are willing to grant their patient’s request for euthanasia. These patients have the option to apply to the “Expertise Centre Euthanasia,” where independent doctors assess whether euthanasia can be granted, and if so, they are involved in carrying out this request (sometimes including organ donation). Another issue, that still requires attention, is in cases where the request for euthanasia is based on psychiatric suffering. For these patients it is even harder to find a physician (psychiatrist) willing to facilitate euthanasia and a donor hospital where organ donation can take place. Although DCD-V fulfils the patient’s explicit wish and has a promising contribution to the donor pool, it is wrong to assume that the practice is universally supported in the Netherlands, especially for the condition of psychiatric suffering.

In the UK euthanasia is illegal, though this does not mean individuals are necessarily prosecuted if they provide assistance to their loved one’s death. For those in the UK who strongly wish for assisted dying but with clearer legal safeguards for their family and friends, they often make the journey to Dignitas in Switzerland [5]. In the UK the Scottish Parliament [6] and the Channel Island of Jersey [7] are both considering the introduction of medical assistance in dying. If it comes, the example from other nations, is that the donation and transplantation community will need to be ready for patients also making requests for DCD-V.

Susanna et al. paper may provide us with the benchmarking science, the ethical challenge is harder to resolve.

## Data Availability

The raw data supporting the conclusions of this article will be made available by the authors, without undue reservation.

## References

[1] Susanna C Van Dijk N de Jongh W Verberght H Van Mook WNKA Bollen J Promising Results of Kidney Transplantation From Donors Following Euthanasia During 10 Year Follow-Up: A Nationwide Cohort Study. Transpl Int (2024). In press. 10.3389/ti.2024.13142 PMC11528710 39494307

[2] Council of Europe. International Figures on Organ Donation and Transplantation – 2022. Transpl Newsl (2023). Available from: https://freepub.edqm.eu/publications/PUBSD-87/detail. Accessed September 12, 2024.

[3] Woodyatt L D’Costa R Plas M Radford S. P9.3: Australia’s First Experience of Organ Donation After Voluntary Assisted Dying - A Experiential Review. Transplantation (2023) 107(10S1):104. 10.1097/01.tp.0000993640.03842.7b

[4] Weiss MJ Dupras-Langlais M Lavigne M-J Lavigne S Martel A-C Chaudhury P. Organ Donation After Medical Assistance in Dying: A Descriptive Study From 2018 to 2022 in Quebec. Can Med Assoc J (2024) 196(3):E79–E84. 10.1503/cmaj.230883 38286494 PMC10833101

[5] The Guardian. UK Membership of Dignitas Soars by 24% as Assisted Dying in Scotland Moves Closer (2024). Available from: https://www.theguardian.com/society/2024/mar/28/dignitas-uk-membership-assisted-dying-scottish-parliament-bill. Accessed September 12, 2024.

[6] The Scottish Parliament. Assisted Dying for Terminally Ill Adults (Scotland) Bill (2024). Available from: https://www.parliament.scot/bills-and-laws/bills/assisted-dying-for-terminally-ill-adults-scotland-bill. Accessed September 12, 2024.

[7] Government of Jersey. Assisted Dying in Jersey. Available from: https://www.gov.je/Caring/AssistedDying/pages/assisteddying.aspx. Accessed September 12, 2024.

